# Supplementary material for: Electromagnetic Interference Shield of Highly Thermal-Conducting, Light-Weight, and Flexible Electrospun Nylon 66 Nanofiber-Silver Multi-Layer Film
Source: Polymers (Basel). 2020 Aug 11;12(8):1805. doi: 10.3390/polym12081805 (PMC7465716; doi:10.3390/polym12081805)
Supplement: Supplementary file 1 [file polymers-12-01805-s001.pdf]

polymers

Supplementary Materials

Electromagnetic interference shield of highly thermal-conducting, light-weight, and flexible electrospun nylon 66 nanofiber-silver multi-layer film

Jaeyeon Kim [1,†], Suyeong Lee [1,†], Changho Kim [1,†], Yeongcheol Park [1], Mi-Hyun Kim [2], and Jae Hun Seol [1,*]

[1] School of Mechanical Engineering, Gwangju Institute of Science and Technology (GIST), Buk-gu, Gwangju 61005, Korea; bestkjy0426@gmail.com (J.K.); silver4fox@gm.gist.ac.kr (S.L.); umtoss530@gist.ac.kr (C.K.); young13id@gist.ac.kr (Y.P.);

[2] ICT Materials & Components Research Laboratory, ETRI, 218 Gajeong-ro, Yuseong-gu, Daejeon 305-700, Korea; kmhyun@etri.re.kr (M.-H.K);

* Correspondence: jhseol@gist.ac.kr

† These authors contributed equally to this work.

Morphology of the fabricated samples: E-0, N-0, E-50, and N-50

Figure S1 shows plan-view SEM images of the fabricated samples as listed in Table 1 in the Manuscript. The samples, which consist of only nylon 66 without Ag layers, *i.e.*, E-0 and N-0, are shown in Fig. S1(a) and (b). Also, SEM images of the Ag layers in E-50 and N-50 are presented in Fig. 3(d) in the Manuscript and Fig. S1(c), respectively. Although the Ag layer in E-50 was annealed and flattened after hot-pressing, the porous and anisotropic structure of the nylon 66 mat was still preserved as shown in Fig. 3(d). In addition, the non-porous structure of the nylon 66 film remained intact after the Ag deposition and subsequent hot-pressing process as shown in Fig. S1(c). However, there were some imperfections such as pores and non-uniform thickness.

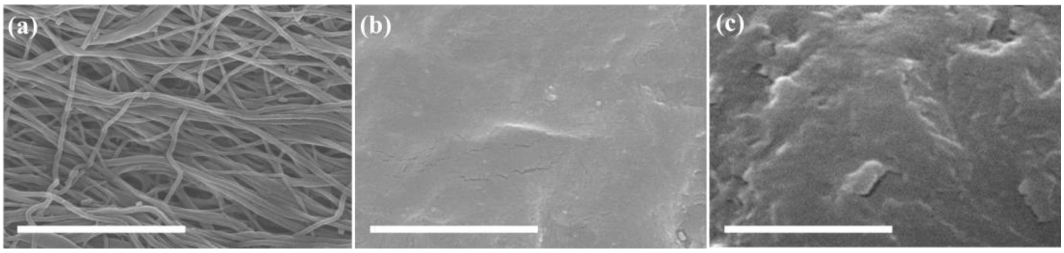

Figure S1. SEM images of the nylon 66 and/or the deposited Ag layers in (a) E-0, (b) N-0, and (c) N-50. The scale bars are 2 μm.

Pore size distributions of ethanol-treated and hot-pressed mats with and without the Ag-deposition

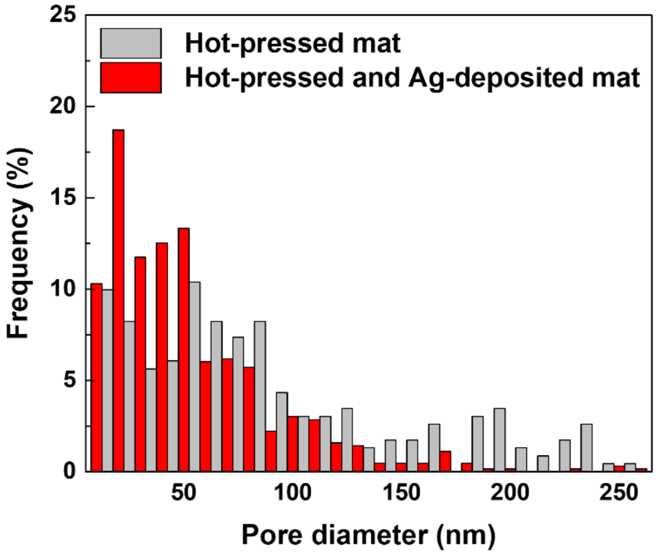

Figure S2. Pore size distributions of the ethanol-treated and then hot-pressed mats without (gray) and with (red) the Ag-deposition. The average pore diameter of the former and latter is 100.0 and 58.6 nm, respectively. The portion of pores with smaller size increases during the metal deposition process.

DSC analysis

To characterize the effect of the hot-pressing temperature on the crystallization of nylon 66 electrospun mats, DSC measurements were conducted while ramping temperatures from 40 to 300 °C at a heating rate of 10 °C/min and using a nitrogen purge gas. Figure S3(a) shows the DSC thermographs of the electrospun mats, which were hot-pressed under different temperatures. During the heating runs, broad endotherm peaks were observed at low temperatures because of solvent evaporation[1]. Table S1 shows the analysis results of the DSC measurements, which summarize the two endothermic temperatures, melting enthalpy, and crystallinity of the electrospun mats. The electrospun mats have two melting endotherms without cold crystallization, which indicates that those melting endotherms originate from imperfect α-phase formed during the electrospinning process [1]. In addition, one of the endotherm peaks disappeared after hot-pressing above 120 °C. The crystallinity was calculated based on the ratio of melting enthalpy (ΔH_m) to that of theoretical 100% crystalline nylon 66 ($\Delta H_m^0 = 200.8$ J/g) [2]. Figure S3(b) shows the degree of crystallinity of the mats and film in relation to the hot-pressing temperature. The crystallinity of mats gradually increased with the hot-pressing temperature because polymeric molecules are better recrystallized at higher annealing temperature [3,4]. Therefore, the mats hot-pressed at higher temperature have higher crystallinity values. Moreover, the film was hot-pressed only at 160 °C, and it shows crystallinity similar to the crystallinity of a mat hot-pressed at 70–120 °C.

Table S1. Two melting endotherms, melting enthalpy, and crystallinity of the DSC measurements

Sample	T_{m1} (°C)	T_{m2} (°C)	ΔH_m (J/g)	Crystallinity (%)
**As-spun**	260.56	269.74	80.22	38.9
**Ethanol-treated**	260.35	267.60	87.82	42.6
**Ethanol-treated/hot-pressed at 40 °C**	260.64	265.04	84.01	40.8
**Ethanol-treated/hot-pressed at 70 °C**	262.66	268.02	87.96	42.7
**Ethanol-treated/hot-pressed at 120 °C**		267.06	88.45	42.9
**Ethanol-treated/hot-pressed at 160 °C**		265.93	95.20	46.2

**Ethanol-treated/hot-pressed at 200 °C**	266.02	97.63	47.4

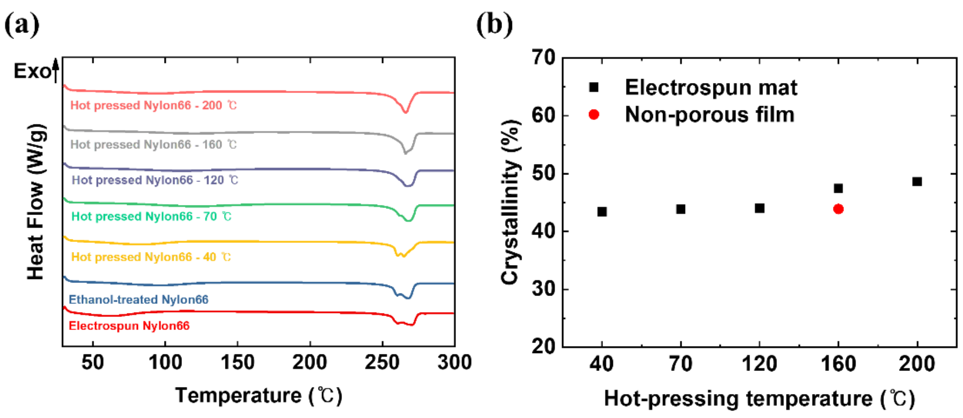

Figure S3. DSC measurement results of the electrospun nylon 66 mats and film. (a) DSC thermographs of the electrospun mats produced from as-spun, ethanol-treated, and ethanol-treated mats hot-pressed at 40, 70, 120, 160, and 200 °C. (b) Measured crystallinity using DSC for ethanol-treated hot-pressed electrospun mat (black square) and ethanol-treated hot-pressed non-porous film (red circle) as a function of the hot-pressing temperature. The crystallinity of non-porous film was hot-pressed only at 160 °C. The measurements were performed with a heating rate of 10 °C/min and a nitrogen purge gas.

Contribution of electrical conductivity to SE$_A$

To investigate the relationship between the electrical conductivity and the absorption of the shield, electromagnetic interference shielding effectiveness (EMI SE) values, resulting from the different electrical conductivity of the deposited Ag layer in N-50 and E-50, were numerically calculated using COMSOL, as shown in Fig. S4. Because there exists a dependence of the absorption loss of an EM wave of a material on its electrical conductivity [5], the electrical conductivity of Ag is one of the factors which enhance the absorption during the multiple reflections. However, although the difference in electrical conductivity was considerable, as shown in Fig. 3 (Manuscript), the increase in absorption SE is insignificant. Therefore, the lower electrical conductivity of E-50 is also large enough to cause multiple reflections.

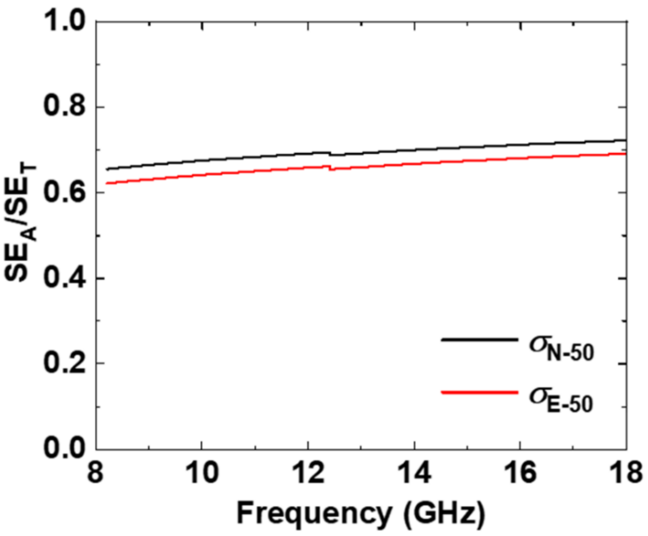

Figure S4. Numerically calculated SE$_A$/SE$_T$ of a multi-layered structure with applied electrical conductivity of Ag layers in N-50 (black continuous line) and E-50 (red continuous line) on the X- and K$_u$-bands.

Mesh configuration for numerical calculation

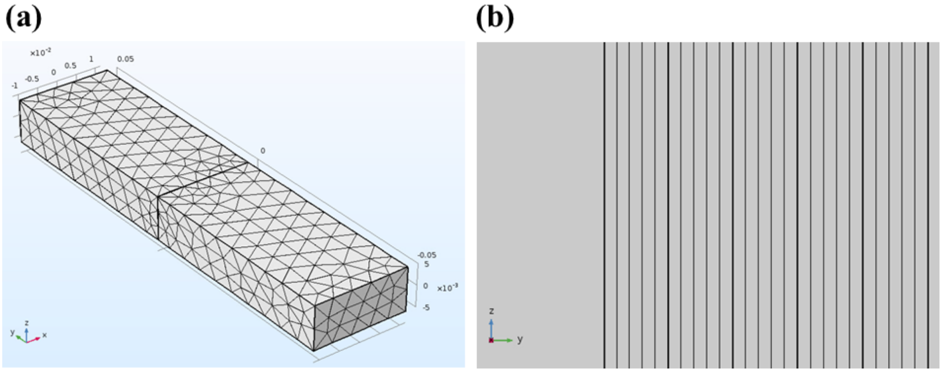

Figure S5. Mesh configurations for numerical calculation using COMSOL. (a) "Normal mesh" type was applied as a waveguide, including a sample at the center of the waveguide. (b) Enlarged mesh configuration in the central region of the waveguide.

Analytical calculation of EMI SE

The SE and the contribution of multiple inter-layer reflections of N-50 were analytically calculated following the transfer-matrix method [6]. According to the transfer-matrix method, the reflection coefficient and transmission coefficient of N-50 could be obtained using recursive propagation matrices corresponding to each layer. The EM wave in a rectangular waveguide is a plane wave and incident normally to the interface, and the electric field at position z can be described as

$$E(z) = \ E_0^+ e^{-\gamma z} + E_0^- e^{\gamma z} = \ E^+(z) + E^-(z), \tag{S1}$$

where E_0^+ and E_0^- are the arbitrary constant vectors that satisfy $\hat{z} \cdot E_0^\pm = 0$ and γ is the propagation constant, defined as $\gamma = \sqrt{j\omega\mu(\sigma + j\omega\varepsilon)}$ where μ, σ, ε, and ω are the permeability, conductivity, permittivity, and angular frequency of the incident EM wave, respectively. Figure S6(a) shows a schematic cross-sectional view representing electric fields which propagate in the ith layer of the multi-layer structure. Therefore, the electric field of the ith interface can be written as $E_i = E_i^+ + E_i^-$, where E_i^+ and E_i^- are the electric fields of E_i propagating forward ($\hat{z}$) and backward (-$\hat{z}$), respectively. Assuming homogenous and isotropic materials, the intrinsic impedance of the ith layer is $\eta_i = \sqrt{j\omega\mu_i/(\sigma_i + j\omega\varepsilon_i)}$. Then, the reflection coefficient (q_i) and transmission coefficient (p_i) at the ith interface are defined with the intrinsic impedance of ith and $(i\text{-}1)^{\text{th}}$ layers as follows:

$$E(z) = \ E_0^+ e^{-\gamma z} + E_0^- e^{\gamma z} = \ E^+(z) + E^-(z), \tag{S2}$$

$$E(z) = \ E_0^+ e^{-\gamma z} + E_0^- e^{\gamma z} = \ E^+(z) + E^-(z), \tag{S3}$$

From the transfer-matrix theory, the relation between $E_i^\pm$ and $E_{i+1}^\pm$ can be described as

$$\begin{pmatrix} E_i^+ \\ E_i^- \end{pmatrix} = M_i \begin{pmatrix} E_{i+1}^+ \\ E_{i+1}^- \end{pmatrix} = \frac{1}{p_i} \begin{pmatrix} e^{\gamma_i t_i} & q_i e^{\gamma_i t_i} \\ q_i e^{\gamma_i t_i} & e^{\gamma_i t_i} \end{pmatrix} \begin{pmatrix} E_{i+1}^+ \\ E_{i+1}^- \end{pmatrix}, \tag{S4}$$

where M_i, γ_i, and t_i are the transfer-matrix, the propagation constant, and the thickness of the ith layer. The structure of N-50 is composed of four Ag layers (*i* = 2, 4, 6, 8) and five nylon 66 layers (*i* = 1, 3, 5, 7, 9), placed between two semi-infinite media of air (*i* = 0, 10) as shown in Fig. S6(b). The total transfer matrix of N-50 (M_{N-50}) was calculated by multiplying the transfer-matrices for each layer,

$$\begin{pmatrix} E_1^+ \\ E_1^- \end{pmatrix} = \prod_{i=1}^{9} M_i \begin{pmatrix} E_{10}^+ \\ E_{10}^- \end{pmatrix} = \prod_{i=1}^{9} M_i \frac{1}{p_{10}} \begin{pmatrix} E'^+_{10} \\ 0 \end{pmatrix} = M_{N-50} \begin{pmatrix} E'^+_{10} \\ 0 \end{pmatrix} \tag{S5}$$

$$M_{N-50} = \frac{1}{p_{10}} \prod_{i=1}^{9} M_i = \begin{pmatrix} a & b \\ c & d \end{pmatrix}, \tag{S6}$$

where E'^{+}_{10} is the electric field transmitted through N-50 toward the air. Using the definition of a scattering matrix, the S-parameters of N-50 can be calculated using the components of M_{N-50} as

$$\mathrm{S}_{11} = \frac{E_1^-}{E_1^+} = \frac{\mathrm{c}}{\mathrm{a}} \tag{S7}$$

$$\mathrm{S}_{21} = \frac{E'^{+}_{10}}{E_1^+} = \frac{1}{\mathrm{a}} \tag{S8}$$

according to Eq. (1–3) in the Manuscript, the SE can be calculated from these results as follows:

$$\mathrm{SE_R} = -10\log(1 - \left|\frac{\mathrm{c}}{\mathrm{a}}\right|^2) \tag{S9}$$

$$\mathrm{SE_A} = -10\log(\left|\frac{1}{\mathrm{a}}\right|^2 / (1 - \left|\frac{\mathrm{c}}{\mathrm{a}}\right|^2)) \tag{S10}$$

$$\mathrm{SE_T} = \mathrm{SE_A} + \mathrm{SE_R} \tag{S11}$$

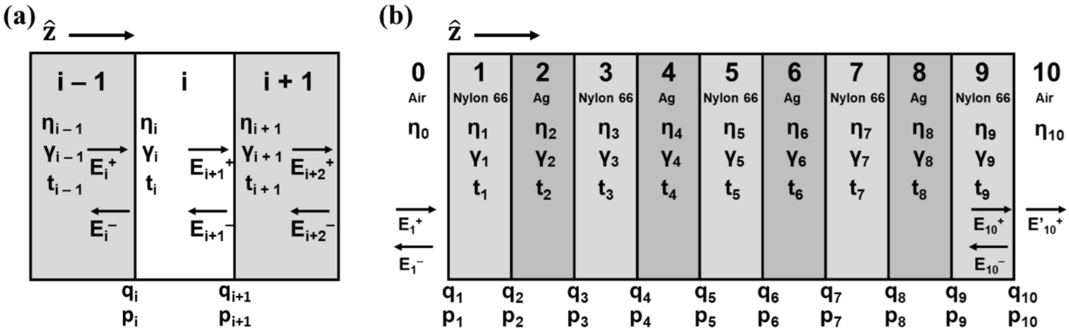

Figure S6. Schematic cross-sectional diagrams of ith layer in a multi-layer structure and N-50. (a) The ith interface refers to the left interface of the ith layer, and the E_i^+ and E_i^- are forward ($\hat{z}$) and backward ($-\hat{z}$) electric fields on the left side of the ith interface. Moreover, q_i and p_i refer to the reflection coefficient and transmission coefficient at the ith interface. (b) Cross-section schematics of N-50. The intrinsic impedance, propagation constant, and thickness of the ith layer are expressed as η_i, γ_i, and t_i, respectively, when i is 0 to 10.

Using these processes, the SE of N-50 was calculated using the measured electrical conductivity of Ag in N-50, other material properties of Ag and nylon 66 from the literature [7,8], and MATLAB software. Additionally, the contribution of inter-layer multiple reflections to $\mathrm{SE_A}$ was obtained by subtracting the penetration loss when the EM wave passes through a material without reflection. The penetration loss contributes to $\mathrm{SE_A}$ by attenuating the EM waves when it passes through the material. According to the shielding theory [9], the penetration loss is defined as

$$\mathrm{SE_{A,penetration}}\ (\mathrm{dB}) = 20\log\left|e^{\sum_{i=1}^{9}\gamma_i t_i}\right| \tag{S12}$$

Influence of hot-press process on the porosity of E-50

As for the porosity calculations, the porosity (ϕ) was calculated from a true density (ρ_{true}) and the density including pores (ρ), *i.e.*, $\phi = (\rho_{true} - \rho)/\rho_{true}$, where ρ is calculated from the ratio of its mass to the volume. Figure S7 shows the porosity values of the as-spun, the firstly hot-pressed mat, and E-50. The firstly hot-pressed mat and E-50 were made of three as-spun mats and five firstly hot-pressed mats through the first and second hot-pressing processes, respectively. The obtained porosity values of the as-spun mat, the firstly hot-pressed three mat, and E-50 were 74.3, 70.0, and 27.7%, respectively. The porosity decreased drastically by a factor of 2.5 after the second hot-pressing process. The thickness values of the feeler gauges, which were used for the first and second hot-pressing processes, were 30 and 100 μm, respectively. Although the thicker feeler gauge was used for the second hot-pressing process, the effect of hot-pressing was more significant because five firstly hot-pressed mats were hot-pressed together.

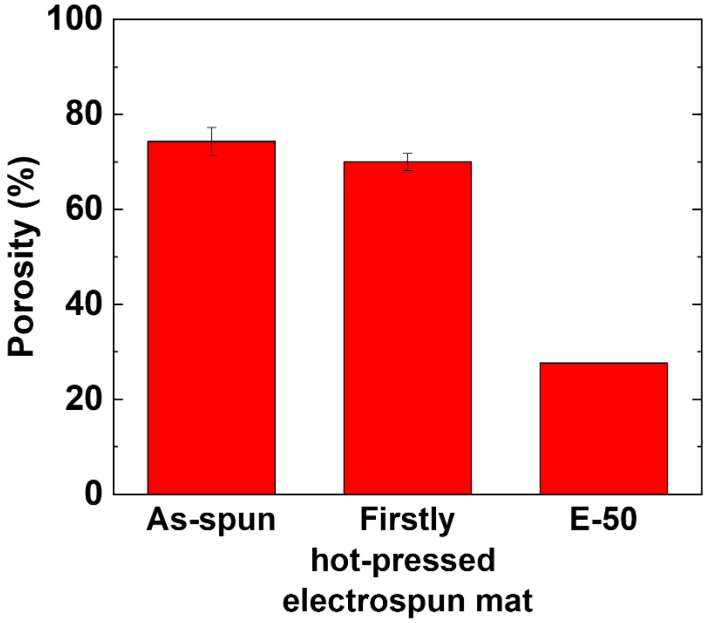

Figure S7. Porosity of as-spun, firstly hot-pressed electrospun mat, and E-50. Notably, the thickness of E-50 was enough to be measured, which made the error bar of E-50 unnoticeable.

References

1. Guerrini, L.M.; Branciforti, M.C.; Canova, T.; Bretas, R.E.S. Electrospinning and characterization of polyamide 66 nanofibers with different molecular weights. *Mater. Res.* **2009**, *12*, 181–190.
2. Mark, J.E. *Physical properties of polymers handbook*; AIP Press: New york, NY, USA, 1996; ISBN 978-0-387-69002-5.
3. Aly, A.A. Heat Treatment of Polymers: A Review. *Int. J. Mater. Chem. Phys.* **2015**, *1*, 132–140.
4. Simal, O.; Martin, A.R. Structure of Heat-Treated Nylon 6 and 6.6 Fibers. I. The shrinkage mechanism. *J. Appl. Polym. Sci.* **1997**, *68*, 441–452.
5. Kuruvilla, J.; Runcy, W.; Gejo, G. *Materials for Potential EMI Shielding Applications: Processing, Properties and Current Trends*; Elsevier Science, 2019; ISBN 9780128175910.
6. Orfanidis, S.J.; Sophocles, J.O. Electromagnetic Waves and Antennas. *Media* **2003**, *2*, 313–321.
7. Elmajid, H.; Terhzaz, J.; Ammor, H.; Chaibi, M.; Mediavilla Sánchez, Á. A new method to determine the complex permittivity and complex permeability of dielectric materials at X-band frequencies. *Int. J. Microw. Opt. Technol.* **2015**, *10*, 34–39.

8. Wu, W.M.; Njoku, C.C.; Whittow, W.G.; Zagoskin, A.M.; Kusmartsev, F. V.; Vardaxoglou, J.C. Studies of permittivity and permeability of dielectric matrix with cuboid metallic inclusions in different orientations. *J. Adv. Dielectr.* **2014**, *04*, 1450032.
9. Transactions, I.; Electromagnetic, O.N. Shielding theory and practice - Electromagnetic Compatibility, IEEE Transactions on. **1988**, *30*, 187–201.
